# COVID-19 and Genitourinary Tract: A Retrospective Study in the Tertiary Care Center

**DOI:** 10.7759/cureus.27153

**Published:** 2022-07-22

**Authors:** Rakesh Gupta, Mukteshwar Kumar, Ishwar Ram Dhayal

**Affiliations:** 1 Urology and Renal Transplant, Dr. Ram Manohar Lohia Institute of Medical Sciences (Dr RMLIMS), Lucknow, IND

**Keywords:** sars-cov-2, corona virus, male genital system, urinary tract, luts, covid -19

## Abstract

Introduction

COVID-19 pandemic has spread across the globe in the last two years and COVID-19 pneumonia is its typical presentation. Coronavirus (SARS-CoV-2) has the potential to affect extrapulmonary sites like the involvement of the urinary tract and male genital organs.

Objectives

This single institutional retrospective observational study aimed to study the effects of COVID-19 on the lower urinary tract (LUT) and male genital system.

Methods

COVID-19 effect on the genitourinary tract was studied in a retrospective observational setting in a tertiary care setting from March 2020 to December 2021 consisting of 110 patients. After informed consent from patients, retrospective data collection was included in a repository. Presenting features related to the genitourinary tract were noted and basic biochemical profiles like CBC, RFT, LFT, urine analysis, and urine culture for bacterial sensitivity were performed in all the patients affected by COVID-19. High-resolution ultrasound was sparingly used according to the clinical presentation of these COVID-19 patients.

Results

A total of 110 patients 95 males and 15 females were included in this study. De novo LUT symptoms were present in 10 (9.09%) patients and acute worsening of these was seen in three patients. Scrotal discomfort was the most common symptom among men found in five (5.26%) patients and frequency of urine was the overall most common symptom found in 13 (12.38%) patients also having two female patients. Among biochemical findings, microscopic hematuria in 68 (61.81%), pyuria in 72 (65.45%), and raised blood urea were observed in 41 (37.27%) patients with COVID-19. Ultrasound findings revealed acute epididymal-orchitis in 3 (3.15 %) and acute orchitis/epididymitis in one (1.05%) case, respectively.

Conclusions

COVID-19 patients do have the chance of developing the involvement of the urinary tract and male genital system and the clinicians should be aware of this so that they can manage these patients accordingly.

## Introduction

Nothing has threatened and ravaged humanity like the COVID-19 pandemic in the last two and half years. Its effects are global and most importantly economic collapse is most tangibly palpable [1-4]. Like all fields and specialties urology is also facing COVID-19-related challenges. Severe acute respiratory syndrome coronavirus 2 (SARS-CoV-2) is the causative virus for this Coronavirus Disease 2019 and the WHO declared this novel coronavirus pneumonia a pandemic on March 11, 2020 [5]. Chest x-ray and computed tomography detect early pulmonary changes in COVID-19 pneumonia and are quite useful in the surveillance of the disease [6,7]. Involvement of the urinary tract and male genital system produces vague symptoms hence clinicians should pay particular attention to their early detection.

This study aims to develop an in-depth inclination among urologists to develop a mindset regarding these COVID-19-related genitourinary conditions for better management and to promote research in this connection.

## Materials and methods

COVID-19 effect on the genitourinary tract was studied in a retrospective observational setting in a tertiary care setting from March 2020 to December 2021 consisting of 110 patients. The study was carried out at Dr. Ram Manohar Lohia Institute of Medical Sciences Lucknow, India in the Department of Urology and Renal Transplant. Presenting features about the genitourinary tract were noted and basic biochemical profiles like CBC, RFT, LFT, urine analysis, and urine culture for bacterial sensitivity were performed in all the patients affected by COVID-19. High-resolution Ultrasound was sparingly used according to the clinical presentation of these COVID-19 patients. Observations were made based on the data repository. All confirmed cases of COVID-19 during this period were included in this study. Strict follow-up was ensured by the institute control room to garner data and a post- COVID-19 intensive care unit was specially established to manage post-COVID-19 complications. Respiratory physicians and intensivists were at the frontline to manage these patients. Urologists were consulted on patients having signs and symptoms related to the urinary tract and male genital system.

Being a super-specialty tertiary care center data retrieval and record-keeping was meticulous and easily accruable. This retrospective observational study was unsponsored research and informed consent (verbal/written) has been obtained or waived. No identifying information appears in this article about patient identity.

## Results

Data of 110 patients with a predominantly male population 95 (86.36%) were retrospectively included in this observational study from March 2020 to December 2021. Among these 110 patients, 10 (9.09%) patients were having preexisting lower urinary tract (LUT) symptoms. Three patients with de novo LUTs developed worsening of their symptoms post-COVID-19. Scrotal discomfort was the most common symptom among men found in five (5.26%) patients and frequency of micturition was the overall most common symptom found in 13 (12.38%) patients including two female patients. Scrotal pain associated with swelling was encountered in four (4.21%) patients. One patient developed scrotal erythema which was mild and associated with increased blood flow on USG color Doppler. Low flow priapism was noted in one patient, which resolved spontaneously. None of these symptoms were bothersome and required only nonspecific supportive treatment in the form of anti-inflammatory and analgesic drugs and an occasional short course of antibiotics, especially in those having acute epididymal orchitis demonstrated on ultrasound examination. Acute epididymo-orchitis was encountered in three (3.15%) patients and one each had acute epididymitis and acute orchitis (Table 1).

**Table 1:** Symptomatology of COVID-19 cases demonstrating genitourinary involvement

Signs and symptoms	Number of patients	Percentage (%)
Lower urinary tract symptoms (LUTs)		
Frequency	13	12.38
Urgency	09	8.18
Urge incontinence	01	0.90
Nocturia	02	1.81
Hematuria	01	0.90
Urinary retention	01	0.90
Dysuria	02	1.81
Scrotal discomfort	5	5.26 (male cohort)
Scrotal pain/swelling	4	4.21
Scrotal erythema	1	1.05
Mild priapism	1	1.05
De novo LUTs	10	9.09

Other LUTs like urgency, urge incontinence, and acute retention due to hematuria associated with clots, and nocturia were also observed (Table 1). Among the laboratory investigations raised blood urea was observed in 41 (37.27%), microscopic hematuria in 68 (61.81%), and transient pyuria occurred in 72 (65.45%) COVID-19 patients (Table 2). Urine culture came positive for bacteria in 10 (9.09) patients.

**Table 2:** Demographic and investigation characteristics of COVID-19 cases Abbreviations: HPF, high power field.

Variables	Values	Percentage (%)
Age in years (Range)	17-91 (61)	
Sex - Male	95	86.36
Female	15	13.67
Blood Urea (mg/dl)	41	37.27
Urine Red Blood Cells (RBCs/HPF >3)	68	61.81
Urine White Blood Cells (WBCs/HPF >5)	72	65.45
Bacterial Urine Culture positive	10	9.09
Ultrasound findings -Acute epididymo- orchitis	03	3.15
Acute epididymitis	01	1.05
Acute orchitis	01	1.05

## Discussion

It is generally considered that pulmonary involvement is the most common pathologic manifestation of COVID-19 but recent studies also indicate rare involvement of the urinary tract and male genital system. LUTs predominantly storage symptoms are the remarkable complaint in these COVID-19 cases but the exact pathogenesis remains unclear [7,8]. Regarding pathogenesis, it is postulated that the involvement of basal urothelial and luminal cells which express ACE2 receptors leads to genitourinary manifestations [9]. Pain and scrotal discomfort with a radiological demonstration of inflammatory changes in the testis and epididymis is the classic presentation of SARS-CoV-2 infection. Involvement of testis occurs by the hematogenous route and postmortem studies have also confirmed these findings [10]. Involvement of the male genital system may impair fertility but data in this regard are lacking like its long-term effects [11]. Thrombotic complications lead to the development of low flow priapism and it should be promptly treated to prevent long-term consequences [12,13]. Like other viruses, novel coronavirus can also lead to bladder hemorrhage producing hematuria and its implications like clot retention [14]. Microscopic hematuria was observed in 61.81% of COVID-19 patients in our study. The clinicians should be aware of these complications and we propose that more studies with a larger number of patients will better guide us in this regard.

Similarly raised blood urea was seen in 37.27% of patients and acute kidney injury has increased mortality in COVID-19 patients [15]. Unlike the first wave of the corona pandemic now it is well established that involvement of the urinary tract and male genital system occurs in less than 5% of patients and in a few cases the presentations are also important and need proactive management. Simultaneously de novo symptoms and underlying conditions like chronic kidney disease should also be taken into account for proper management. 

Limitations

It is a single institute-based observational study, small sample size, and effects on fertility are not studied hence further studies are required to overcome these limitations.

## Conclusions

There are now sufficient data to suggest that involvement of the urinary tract and male genital system occurs in certain patients (up to 5%) hence clinicians should be aware of these manifestations so that timely management is instituted thereby decreasing complications due to COVID-19. More research especially systemic reviews/meta-analyses will help in increasing our knowledge in this connection regarding the long-term effects of novel coronavirus on the genitourinary tract.
